# Use of Clinical Public Databases in Hidradenitis Suppurativa Research

**DOI:** 10.2196/70282

**Published:** 2025-02-18

**Authors:** Xu Liu, Linghong Guo, Xian Jiang

**Affiliations:** 1 Department of Dermatology West China Hospital Sichuan University Chengdu China; 2 Laboratory of Dermatology, Clinical Institute of Inflammation and Immunology, Frontiers Science Center for Disease-Related Molecular Network West China Hospital Sichuan University Chengdu China

**Keywords:** hidradenitis suppurativa, clinical public databases, disease progression, patient data, HS

## Abstract

In this viewpoint, we argue that recent studies using clinical public databases have revolutionized our understanding of hidradenitis suppurativa (HS), a chronic inflammatory skin condition with significant impacts on patients’ quality of life. Our key messages are as follows: (1) these databases enable large-scale studies integrating genetic, epidemiological, and clinical data, providing crucial insights into HS’s genetic predispositions, comorbidities, and treatment outcomes; (2) findings highlight a strong genetic component, with mutations in the γ-secretase complex playing a key role in HS pathogenesis and shaping targeted therapies; (3) studies also reveal elevated risks for comorbidities like obesity, diabetes, cardiovascular disease, and systemic inflammation in patients with HS, with diet-driven inflammatory pathways potentially exacerbating disease severity; (4) while these databases offer unprecedented research opportunities, limitations such as data representativeness and quality must be considered; (5) nonetheless, their benefits outweigh potential drawbacks, allowing the identification of rare comorbidities, disease progression patterns, and personalized treatment strategies; and (6) increased funding for HS research is crucial to harness these databases’ full potential, develop targeted therapies, and ultimately improve patient outcomes. As HS’s impact is disproportionate to current research investments, we believe advocating for more resources and addressing database limitations will be key to advancing HS understanding and care.

Hidradenitis suppurativa (HS) is a debilitating chronic inflammatory skin condition that significantly impacts patients’ quality of life. Despite its profound effects, the pathogenesis of HS remains poorly understood [1]. In this viewpoint, we aim to highlight how recent advancements in clinical public databases have provided researchers with a powerful tool to explore the genetic, epidemiological, and clinical aspects of HS (Table 1). We argue that these databases have enabled large-scale studies integrating diverse patient data, yielding crucial insights into the disease’s genetic predispositions, comorbidities, and treatment outcomes [2].

**Table 1:** Recent studies on hidradenitis suppurativa (HS) using clinical public databases.

Title	Year	Journal	Conclusion	Databases
Genetic Susceptibility to Hidradenitis Suppurativa and Predisposition to Cardiometabolic Disease [3]	2024	*JAMA Dermatology*	Explored the genetic susceptibility of HS to cardiovascular and metabolic diseases	UK Biobank
Association Between Hidradenitis Suppurativa and Gout: A Propensity Score-Matched Cohort Study [4]	2024	*Dermatology*	Found an association between HS and gout	TriNetX Research Network
A History of Asthma Is Associated With Susceptibility to Hidradenitis Suppurativa: A Population-Based Longitudinal Study [5]	2023	*Archives of Dermatological Research*	Found a correlation between a history of asthma and susceptibility to HS	Clalit Health Services
Hidradenitis Suppurativa and the Risk of Myocardial Infarction, Cerebrovascular Accident, and Peripheral Vascular Disease: A Population-Based Study [6]	2023	*Archives of Dermatological Research*	Investigated the risk of myocardial infarction, stroke, and peripheral vascular disease in patients with HS	Clalit Health Services
Hidradenitis Suppurativa and Rheumatoid Arthritis: Evaluating The Bidirectional Association [7]	2021	*Immunologic Research*	Evaluated the bidirectional association between HS and rheumatoid arthritis	Clalit Health Services
Association of Birth Weight, Childhood Body Mass Index, and Height With Risk of Hidradenitis Suppurativa [8]	2020	*JAMA Dermatology*	Studied the relationship between birth weight, childhood BMI, and height with the risk of HS	Danish National Patient Register
Global Hidradenitis Suppurativa COVID-19 Registry: A Registry to Inform Data-Driven Management Practices [9]	2020	*British Journal of Dermatology*	Explored data-driven management practices through the Global Hidradenitis Suppurativa COVID-19 Registry	Global Hidradenitis Suppurativa COVID-19 Registry
Comparing Cutaneous Research Funded by the National Institute of Arthritis and Musculoskeletal and Skin Diseases With 2010 Global Burden of Disease Results [10]	2014	*PLoS One*	Compared skin research funded by the National Institute of Arthritis and Musculoskeletal and Skin Diseases with the 2010 Global Burden of Disease results	National Institute of Arthritis and Musculoskeletal and Skin Diseases Database and the Global Burden of Disease

One of our key messages is that studies using clinical public databases have revealed a strong genetic component in HS. By analyzing data from diverse populations, researchers have identified genetic factors, such as mutations in the γ-secretase complex, that predispose certain individuals to develop HS [3,11]. We believe these genetic insights are not only expanding our understanding of HS pathogenesis but also shaping the development of targeted therapies [11]. Furthermore, we emphasize that clinical databases have shed light on the complex relationship between HS and various comorbidities. Studies have shown that patients with HS have a significantly higher risk of developing obesity, diabetes, cardiovascular disease, and even gout [4,6,12,13]. Notably, diet-driven inflammatory pathways, particularly those involving interleukin-17 and interleukin-1β, have been implicated in exacerbating HS severity [12]. The interplay between obesity, immune checkpoint inhibitors, and psoriasiform eruptions in HS further highlights the role of shared inflammatory pathways in disease exacerbation [13]. These findings underscore our view that a holistic approach to managing HS is needed, addressing both the dermatological and broader health risks faced by patients.

While we believe the insights gained from clinical public databases are invaluable, it is essential to consider potential limitations and counterarguments. One concern is the representativeness of the data within these databases. We argue that ensuring diverse and inclusive patient populations is crucial to avoid bias and generate findings applicable to the broader HS community [7]. Additionally, the quality and consistency of data entry across different health care settings may vary, potentially impacting the reliability of the results [5]. Furthermore, biases may exist in terms of the ethnic and geographic diversity of the data, which may skew the results for certain populations. We believe expanding the scope of these databases to include more diverse patient groups will be essential to ensure that the findings are broadly applicable and not limited by sample biases.

Despite these challenges, it is our view that the benefits of using clinical public databases in HS research far outweigh the limitations. These databases provide access to large, diverse patient populations and enable the identification of rare comorbidities and treatment outcomes that may not be apparent in smaller clinical settings. Moreover, longitudinal data captured in these databases allow researchers to study disease progression and identify early diagnostic markers, paving the way for timely interventions and personalized treatment plans [8]. We argue that researchers should focus on making improvements to database infrastructure, such as ensuring data consistency and addressing gaps in representation, to maximize their utility for future studies.

As we continue to harness the power of clinical public databases, we believe it is crucial to prioritize HS research and allocate adequate funding. The Global Burden of Disease study has highlighted the significant impact of HS on patients’ lives, yet research funding for HS remains disproportionately low compared to other skin diseases [9,10]. We argue that increased investment in HS research will accelerate the development of targeted therapies and improve patient outcomes. Public health initiatives aimed at increasing awareness of HS and its comorbidities, along with funding to support further research, will be vital to driving innovation and improving patient care.

In conclusion, it is our view that clinical public databases have revolutionized HS research by providing unprecedented access to large-scale, diverse patient data. The insights gained from these databases have deepened our understanding of the genetic susceptibility, comorbidities, and treatment outcomes associated with HS. As we move forward, we believe it is essential to address the limitations of these databases, ensure inclusive patient representation, and advocate for increased funding for HS research. By harnessing the full potential of clinical public databases, we can unlock new avenues for personalized medicine and ultimately improve the lives of individuals affected by this challenging condition.

## Abbreviations

HS: hidradenitis suppurativa

## Data Availability

No new data were generated or analyzed in support of this viewpoint.
